# Book Notices

**Published:** 1891-03

**Authors:** 


### Book Notices.

A Text-Book of Comparative Physiology for Students and practitioners of Comparative (veterinary) Medicine. By Wesley Mills, M. A., M. D., D. V. S., Montreal, Canada. With 476 illustrations. New York: D. Appleton & Co.: 1880: pp. 636.

This work, as compared with the author's other book on "Animal Physiology," is much more comparative and specialized as regards the domestic amimals. The book is valuable for the general practitioner, though it is intended more particularly for the veterinary student. It deserves the compliment of being called a text-book. B.

---

ANNOUNCEMENT.—E. B. Treat, Pub., N. Y., has in press for early publication the Ninth Yearly issue of the "International Medical Annual."

Its corps of thirty-seven Editors—specialists in their different departments, comprising the brightest and best American, English and French authors—will vie with previous issues in making it even more popular and of more practical value to the Medical Profession.

We have the assurance of some of the best medical practitioners that the service rendered their profession by this Annual cannot be duplicated by any current annual or magazine, and that it is an absolute necessity to every physician who would keep abreast with the continuous progress of practical medical knowledge.

Its index of New Remedies and Dictionary of New Treatment, epitomized in one ready reference volume at the low price of $2.75, make it a desirable investment for the busy practitioner, student and chemist.

---

Electricity in the Diseases of Women—With special reference to the application of strong currents, by G. Bettor Massey, M. D., Philadelphia. Second edition, revised and enlarged. Philadelphia and London. F. A. Davis, publisher, 1890, pp. 240. Price $1.50.

This work proposes to be a complete treatise on the electrical treatment of the diseases of women and it is a very interesting book. All of the good points of the first edition have been preserved and anything new up to date has been included in the present volume. B.

